# Arrowroot Starch Films Cross-Linked with Citric Acid: Susceptibility to Water, Biodegradation, and Tensile Properties

**DOI:** 10.1021/acsomega.6c04185

**Published:** 2026-07-13

**Authors:** Rochelle Moraes Matos, José Antônio da Silva Souza, Jordan Del Nero, Severino Alves, Waldomiro Gomes Paschoal Júnior, Bruno Marques Viegas, Carlos Alberto Brito da Silva, Marcos Vinícius da Silva Paula, Emanuel Negrão Mâcedo

**Affiliations:** † Programa de Pós-Graduação em Engenharia Química, 37871 Universidade Federal do Pará, Belém 66075-110, Brasil; ‡ Programa de Pós-Graduação em Engenharia de Processos, Universidade Federal do Pará, Belém 66075-110, Brasil; § Faculdade de Física, Universidade Federal do Pará, Belém 66075-110, Brasil; ∥ Departamento de Química Fundamental, Universidade Federal de Pernambuco, Recife 51160-070, Brasil; ⊥ Programa de Pós-Graduação em Biotecnologia, Universidade Federal do Pará, Belém 66075-110, Brasil; # Programa de Pós-Graduação em Ciência e Engenharia de Materiais, Universidade Federal do Pará, Ananindeua 67130-660, Brasil; ∇ Faculdade de Física, Universidade Federal do Pará, Ananindeua 67130-660, Brasil

## Abstract

In this study, thermoplastic arrowroot starch (TPA) films cross-linked with citric acid (CA) were prepared using solvent casting method. Different glycerol (25%, 30%, and 35%) and CA (5%, 9%, and 12%) contents were evaluated. The films were characterized using Fourier-transform infrared spectroscopy (FTIR), scanning electron microscopy (SEM), and X-ray diffraction (XRD), as well as by their transparency, thickness, mechanical properties, swelling, moisture content, solubility, water vapor permeability, and soil degradation. FTIR results showed the formation of ester linkages, confirming the cross-linking of arrowroot starch with CA. XRD revealed broad peaks at approximately 20° for all films, characteristic of the amorphous phase of starch. The SEM micrographs revealed more homogeneous and continuous surfaces in the cross-linked films. For films with 30% glycerol, cross-linking increased the tensile strength from 1.74 MPa for the glycerol-free film (TPA-30/CA-0) to 6.60 MPa for the film with 9% CA (TPA-30/CA-9). Additionally, the CA-cross-linked TPA films exhibited less swelling in water for all the evaluated periods. In the soil degradation test, TPA films with CA exhibited slower degradation, with total degradation observed after 18 days. Bread stored with TPA-30/CA-9 and TPA-25/CA-12, exhibited a delay in fungal growth. Thus, based on our results, the TPA-30/CA-9 film presents a suitable potential for use in the food packaging sector.

## Graphical abstract

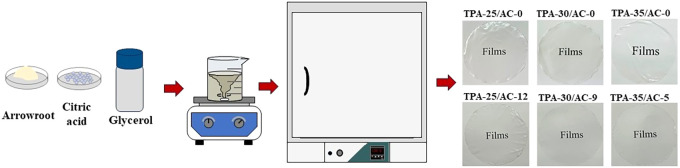

## 1 Introduction

Conventional plastics, derived from petroleum, are in high demand at a rate of over 400 million tons annually and play a fundamental role in various industrial segments.^1–3^ Only 14% of this total waste is recycled, while another 14% is incinerated, and 72% is disposed of in landfills or in the environment.^4–7^ The properties of these materials, such as lightness, strength, ease of transport, and transparency, make them indispensable in various applications.^8^ However, one of the main challenges of these materials is their long degradation time, which leads to their massive and persistent accumulation in the environment.^2,9^ In this context, and in the pursuit of sustainability, the industrial sector and scientific community have been researching alternatives to mitigate these environmental impacts.^10,11^ Biodegradable materials have been established as an option to reduce the environmental impact of petroleum-derived polymers.^12^ Among biodegradable materials, biopolymers are considered excellent candidates for replacing petroleum-derived plastics, as they are extracted from natural and renewable resources.^13^ These materials offer advantages such as high availability, biodegradability, and low cost, in addition to being decomposed by natural biological agents, which helps to prevent soil and water pollution.^3^

Among biopolymers, starch is widely used to develop biodegradable products owing to its abundance, low cost, and biodegradability.^9,12,14^ Starch is a polymer composed of d-glucose units, found in the seeds, tubers, roots, and stems of plants.^15–17^ In its granular form, starch is a mixture of two homopolysaccharides: amylose, a linear molecule, and amylopectin, a highly branched molecule.^15–17^ Amylose constitutes between 15% and 30% of starch and, owing to its linear chain structure, is crucial for the formation of films with good mechanical and barrier properties.^18–20^ Amylopectin, in turn, constitutes the majority of starch, accounting for 70–85% of its composition, and is responsible for the crystallinity of the material.^20^ The proportion and characteristics of these two units are determined by the plant origin, directly influencing the final characteristics of starch.^16^ To process starch, its semicrystalline granular structure must be disrupted and destroyed. The processed material is called thermoplastic starch.^20^ Processing is carried out by adding a plasticizer with continuous heating and shearing, resulting in a homogeneous, continuous viscous phase.^20^ Plasticizers are low-molecular-weight compounds that reduce the interactions between starch chains, providing greater flexibility and processability to the material.^1^ Glycerol is a widely used plasticizer owing to its compatibility, low cost, hydrophilic nature, and ability to reduce the strong intermolecular interactions between starch chains.^9^

In this context, arrowroot (*Maranta arundinacea*), a species native to South America, has emerged as a promising source of starch, with an amylose content of approximately 35%, which is a desirable characteristic for the production of films with improved tensile and barrier properties.^11,21^ The high amylose content favors film formation owing to the ease of amylose chain alignment and approximation, thus facilitating film formation. Classified as an unconventional food species, the use of arrowroot starch directly supports the transition to a circular bioeconomy by utilizing regional biomass resources that do not directly compete with starches used for food purposes, such as corn, potatoes, and cassava.^22,23^ The use of thermoplastic arrowroot starch is highly promising in the food packaging sector; however, despite its advantages, arrowroot starch films share the common limitations of starch, such as low mechanical strength, high permeability, and strong hydrophilic nature, which significantly limit their use in the food packaging sector.^17^

Thus, a solution to these characteristics of starch is its cross-linking with CA. CA is a nontoxic and low-cost cross-linking agent that is widely found in fruits.^17^ Cross-linking occurs through the formation of covalent ester bonds between the carboxyl groups of the acid and the hydroxyl groups of the polysaccharides.^17^ Cross-linking with CA improves the mechanical properties, water resistance, and permeability of starch.^17,24^ Studies have demonstrated that the use of CA in thermoplastic starch films results in significant improvements in their mechanical and barrier properties.^1,2,24,25^ Previous investigations have reported that the tensile strength increases as a function of cross-linking, whereas the deformation tends to decrease owing to reduced molecular mobility.^26^ Additionally, the water vapor permeability is reduced, making the films more efficient for food packaging.^2,17^ These findings confirm that the combination of plasticization and cross-linking constitutes a viable alternative for adjusting the balance between flexibility, rigidity, and barrier capacity, adapting the films to the needs of the packaging sector.^2,17^

In this study, biodegradable arrowroot starch-based films were prepared by solvent casting, using glycerol as a plasticizer at three different concentrations (25%, 30%, and 35%) and CA as a cross-linking agent. The films were cured at 120 °C for 9 min, followed by characterization using Fourier-transform infrared spectroscopy (FTIR), thermogravimetric analysis (TGA), scanning electron microscopy (SEM), and X-ray diffraction (XRD). The mechanical properties, permeation, moisture content, solubility, swelling, transparency, soil degradation, visual appearance, and thickness of the films were also evaluated. Thus, the main objective of this study was to investigate the combined effects of different glycerol and CA contents on the tensile strength, biodegradation, and water susceptibility of TPA films.

## 2 Experimental Section

### 2.1 Materials

Table 1 lists all the reagents used, their chemical compositions, and their suppliers. The reagents were used without purification.

**Table 1:** Description of the Materials

material	composition	supplier
Arrowroot starch	____	Torres Alimentos
Glycerol P.A	C_3_H_8_O_3_	Êxodocientífica
CA P.A	C_6_H_8_O_7_	Neon
Distilled water	H_2_O	

### 2.2 Methodology

#### 2.2.1 Production of Films

The films were obtained using the solution casting method with water as the solvent. Initially, 2 g of arrowroot starch was dispersed in 60 mL of distilled water. Subsequently, glycerol was added at 25%, 30%, and 35% (m/m relative to starch). CA was added to the mixture in different proportions relative to the starch content. The film-forming solution was manually stirred at 85 °C until it reached the gel point. The gelled film-forming solution was poured into 140 mm × 150 mm Petri dishes and dried at 45 °C for 24 h. After drying, the films were removed from the Petri dish and subjected to heat treatment at 120 °C. Subsequently, the films were stored in a desiccator with silica gel for further characterization. Figure 1 shows a representative flowchart of the methodology used for the film preparation.

**Figure 1:**
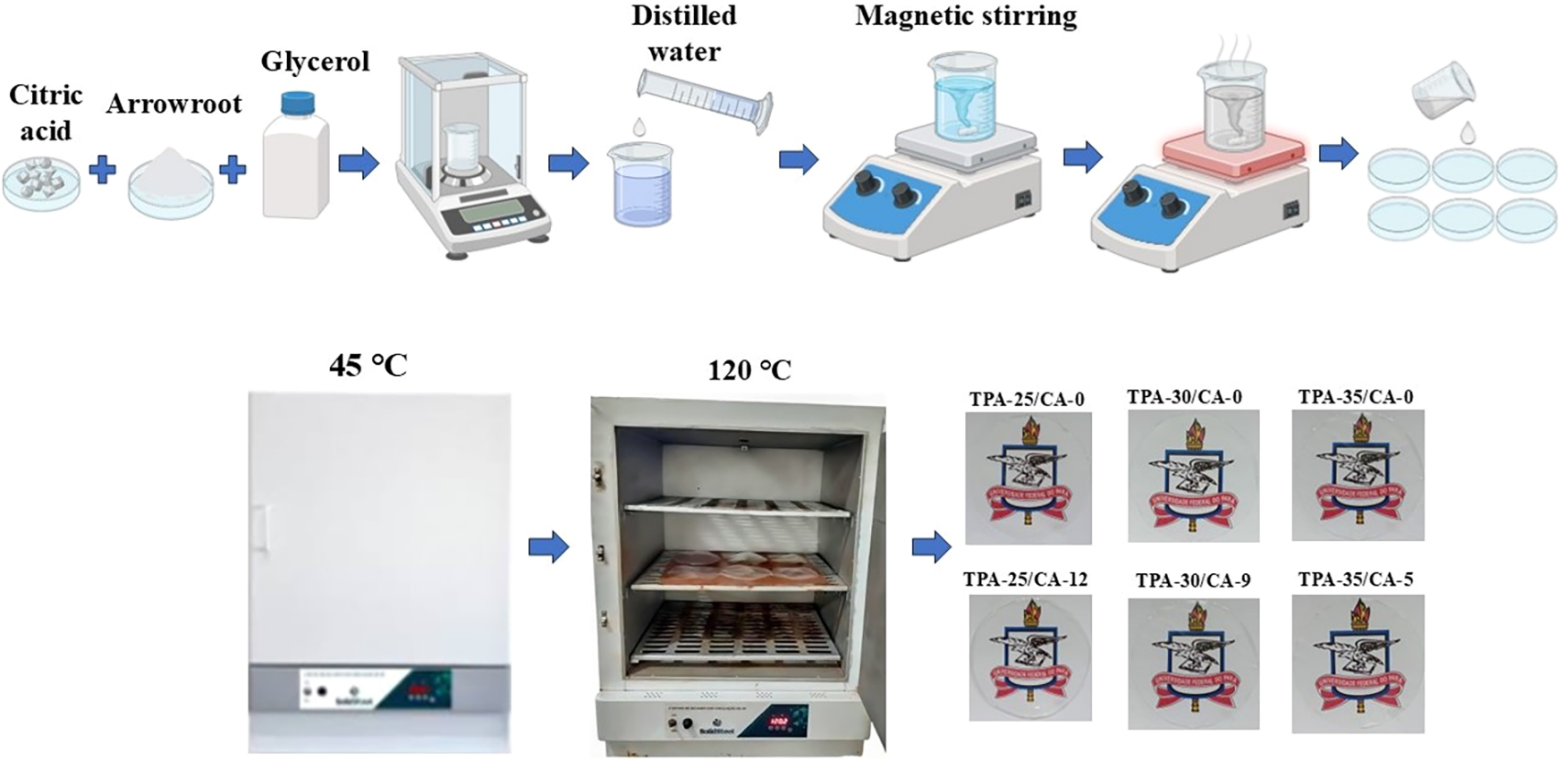
Representative diagram of the methodology used for film preparation.

#### 2.2.2 Swelling in Water

To evaluate the absorption and retention capacity of water at different times, swelling in water was determined using 2 cm × 2 cm samples. The samples were initially weighed (*m*_i_) and immersed in 100 mL of water. After each immersion period, the samples were removed from the water, dried with paper, and weighed again (*m*_f_). The degree of swelling was determined in triplicate for each sample type, based on eq 1.

1 $$\mathrm{SW}\;\left(\%\right)=\left(\frac{m_{f}-m_{i}}{m_{i}}\right)\times 100$$

#### 2.2.3 Cross-Linking Density

The cross-linking density (*v*) of the TPA films was determined using the Flory–Rehner theory. The dry films were weighed (*M*_d_) and immersed in distilled water for different times. After each cycle, excess water was removed from the films using paper and the final mass was recorded (*M*_s_). The volume fraction of the polymer in the swollen gel (v_2_) was calculated using eq ([Disp-formula eq2]).

2 $$v_{2}=\frac{1/P_{p}}{\left(1/P_{p}\right)+\left[\left(M_{s}-M_{d}\right)/\left(M_{d}\times P_{s}\right)\right]}$$

Where *P*_p_ is the density of dry arrowroot starch (0.88 g/cm^3^) and *P*_s_ is the density of water (1.00 g/cm^3^). Then the value of *v* (mol/cm^3^) was obtained using the Flory–Rehner equation (eq 3).^27^

3 $$v=\frac{-\left[\ln\left({1-v}_{2}\right)+v_{2}+Xv_{2}^{2}\right]}{V_{1}\left(v_{2}^{1/3}-\frac{v_{2}}{2}\right)}$$

Where *V*_1_ represents the molar volume of water (18.016 cm^3^/mol) and *X* is the Flory–Huggins interaction parameter for the starch-water system, fixed at 0.48.^27^

#### 2.2.4 Thickness

The thickness of the films was determined using a precision micrometer (resolution of 0.001 mm) by taking five measurements at random points on the surface of each of the three samples.

#### 2.2.5 Fourier Transform Infrared Spectroscopy (FTIR)

FTIR spectra for the TPA films with and without CA were acquired using a spectrophotometer (BRUKER model VERTEX 70 (v)) in attenuated total reflectance (ATR) mode in the range of 4000 to 400 cm^–1^ with 100 scans and a resolution of 8 cm^–1^.

#### 2.2.6 X-ray Diffraction (XRD)

XRD curves were acquired on an X-ray diffractometer (RIGAKU, Smartlab model), Co anode (Ka - 1,789010 Å), with a voltage of 40 kV, current of 35 mA, in the range of 5 to 80°, with an acquisition speed of 0.026° in 27.5 s.

#### 2.2.7 Thermogravimetric Analysis (TGA)

TGA curves for the TPA films with and without CA were acquired using a SHIMADZU DTG-60H instrument in an inert nitrogen environment from room temperature to 600 °C at a heating rate of 10 °C/min.

#### 2.2.8 Scanning Electron Microscopy (SEM)

For SEM image acquisition, the TPA films were first coated with a thin layer of gold using an EMITECH metallizer, model K550 Sputter Coater. Subsequently, SEM images were obtained using a scanning electron microscope from TESCAN, model Mira3 with a field emission gun (FEG) operating at 5 kV.

#### 2.2.9 Moisture Content (MC %)

The gravimetric method was used to determine the moisture content (MC %). In brief, 2 cm × 2 cm films were initially weighed (*m*_i_). The films were then heated at 105 °C for 24 h, after which the films were weighed again (*m*_f_). Three replicates were performed for each film type. To determine the MC%, eq 4 was used.

4 $$\mathrm{MC}\;\left(\%\right)=\left(\frac{m_{i}-m_{f}}{m_{i}}\right)\times 100$$

#### 2.2.10 Solubility in Water

To determine the solubility in water of the films (S %), pieces of films with dimensions of 1.5 cm × 4 cm were heated at 100 °C for 24 h and then weighed (*m*_i_). After drying, the films were immersed in 50 mL water for 24 h. Subsequently, the films were heated at 105 °C for 24 h and weighed again (*m*_f_). Three replicates were performed for each type of film. To determine *S*%, eq 5 was used.^28^

5 $$S\;\left(\%\right)=\left(\frac{m_{i}-m_{f}}{m_{i}}\right)\times 100$$

#### 2.2.11 Water Vapor Permeability

The water vapor permeability (WVP) of the films was determined gravimetrically using the E96–95 method (American Society for Testing and Materials).^29^ Briefly, a cup was filled with anhydrous calcium chloride, and this cup was coated with a film, with a distance of 1–1.5 cm between the film and the anhydrous calcium chloride. The cups were placed in a desiccator with a saturated sodium chloride solution, and the mass of each cup coated with the film was monitored every 24 h for up to 216 h of storage. The WVP determination for each type of film was performed in triplicate, using eq 6 to obtain the WVP of the films.

6 $$WVP=\left[\frac{x}{P_{sat}\left({RH}_{des}-{RH}_{cup}\right)}\right]\left(\frac{\Delta m}{\Delta t\cdot A}\right)$$

Where WVP is the water vapor permeability, *x* is the thickness of the film, *P*_sat_ is the saturation pressure for the water, RH_des_ is the relative humidity in the desiccator, RH_cup_ is the relative humidity in the cup, Δ*m*/Δ*t* is the slope of the mass curve, and *A* is the permeation area of the film.

#### 2.2.12 Transparency

Films with dimensions of 1 cm × 4 cm were inserted into a quartz cuvette. The cuvettes with the films were then inserted into a UV–visible spectrophotometer (SHIMADZU, model UV-1800), and the transmittance at 600 nm was measured. Three replicates were performed for each type of film, and the transparency was determined using eq 7.

7 $$T=\frac{\mathrm{Log}\left(T\%\right)}{x}$$

where, *T* is the transparency, *T*% is the transmittance in %, and *x* is the thickness of the film.

#### 2.2.13 Soil Burial Degradation

Samples measuring 2.5 cm × 2.5 cm were buried in soil, with a humidity of 12%, acquired from a local market (Santa Barbara-Pará state, Brazil) to a depth of 10 cm at ambient temperature. The films were then removed from the soil and monitored macroscopically at 2, 3, 5, 7, 10, 12, 14, 16, 18, and 20 days of burial. Three replicates were performed for each film type.

#### 2.2.14 Application as Bread Packaging

TPA films were used to store artisanal bread without the addition of preservatives. Thermally sealed packages measuring 10 cm × 6 cm were used to contain bread cubes measuring 2 cm × 2 cm × 2 cm. Polyethylene/polypropylene film packaging was used as a control. The occurrence of microorganisms was verified by visual inspection for 7 days.

#### 2.2.15 Tensile Properties

The tensile mechanical properties of the TPA films were determined based on the ASTM D882–18 standard, with modifications, using a Biopdi with a capacity of 50 Kgf. The samples were cut into strips (25 mm × 75 mm), and the analysis was performed at a speed of 5 mm/min. The tensile properties investigated were tensile strength (σ), modulus of elasticity (*E*), and elongation at break (ε), with three replicates performed for each type of film.

#### 2.2.16 Statistical Analysis

Analysis of Variance (ANOVA) using Duncan’s test with a significance level of 5% (*p* < 0.05) was used to determine statistically significant variations between the average values.

## 3 Results and Discussion

### 3.1 Effect of Glycerol Content

To select the films with the best composition, the degree of swelling in water for 24 h was determined for samples with 25%, 30%, and 35% glycerol. For each glycerol content, different CA contents were used (1%, 3%, 5%, 7%, 9%, and 12% w/w relative to the starch). The interaction between the molecular structures of citric acid and starch promotes the formation of a network of cross-links, which confers greater stability to the polymeric matrix. Therefore, evaluating swelling as a function of citric acid content and curing time allowed for the analysis of the dimensional stability of the selected formulations and verification of whether the formed polymeric network could maintain its structural integrity during prolonged periods of contact with water.^30,31^ The compositions with the lowest swelling were selected to determine the curing times. Figure 2 shows the swelling values for the three glycerol contents investigated.

**Figure 2:**
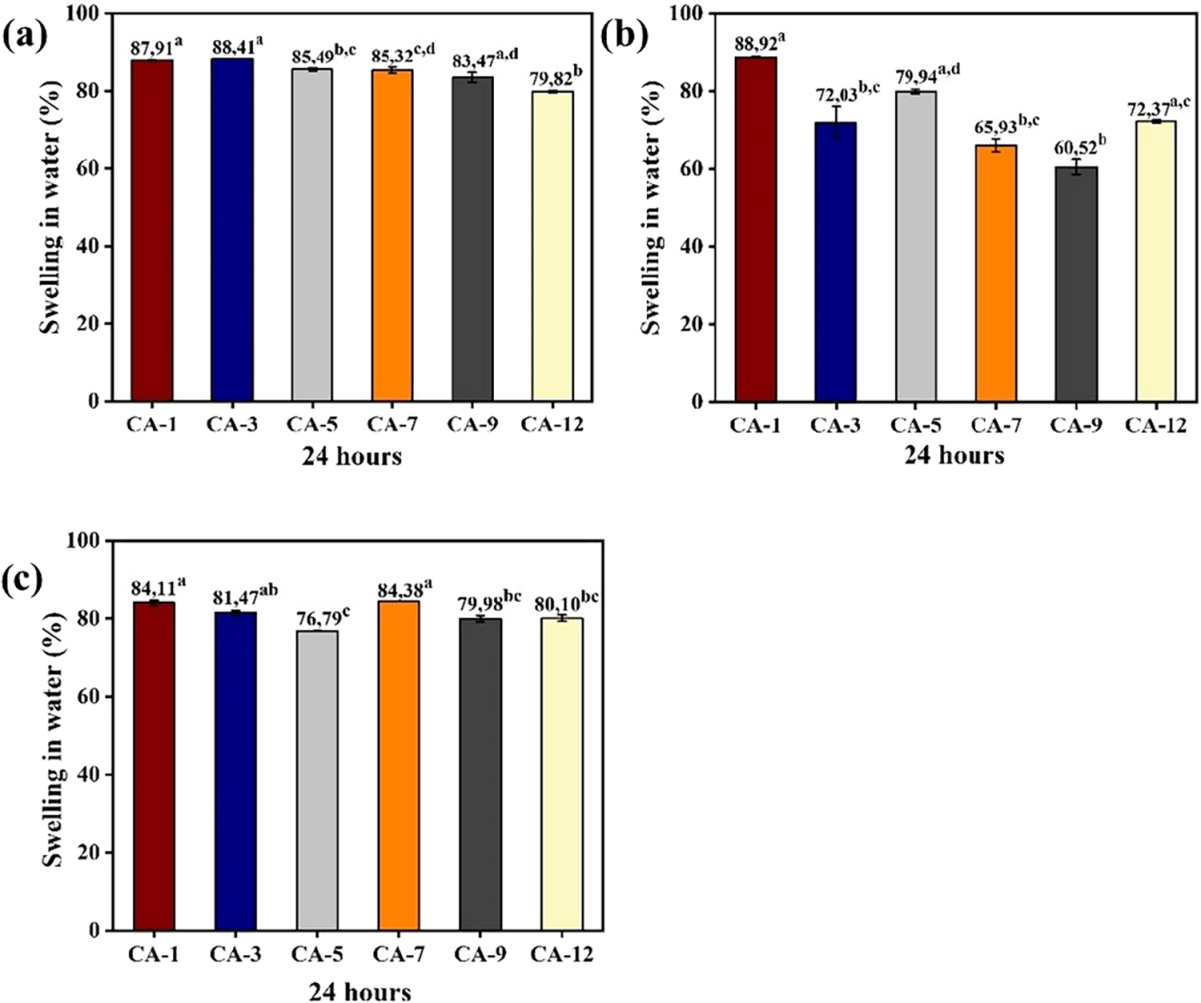
Swelling in water for arrowroot starch films with (a) 25%, (b) 30%, and (c) 35% glycerol contents with different CA contents.

Initially, films with three different glycerol contents (25%, 30%, and 35%) were evaluated with six different CA levels (1%, 3%, 5%, 7%, 9%, and 12%). From this stage, the samples that showed the best results with CA were selected to determine the curing time, which was carried out at 120 °C for 1, 3, 5, 7, 9, and 12 min. The films with 25% glycerol showed statistically significant differences starting from 5% CA, with samples containing 12% CA exhibiting the lowest swelling value and a statistically significant difference compared to the others. The findings of this study corroborate the data presented by Reddy and Yang, who reported that CA concentrations below 5% resulted in relatively low performance in the evaluated Properties.^24^ For films with 30% glycerol, the lowest swelling rate was obtained for the film with 9% CA content. The results for films with 35% glycerol showed that the samples with 5% CA exhibited the lowest swelling. Based on these results, the CA concentrations selected for evaluating the curing time were 12% for films with 25% glycerol, 9% for films with 30% glycerol, and 5% for films with 35% glycerol.

### 3.2 Curing Time

The selected samples (25% glycerol with 12% CA, 30% glycerol with 9% CA, and 35% glycerol with 5% CA) were subjected to different curing times (1, 3, 5, 7, 9, and 12 min). Subsequently, the swelling in water at 24 h was determined for each type of sample (Figure 3).

**Figure 3:**
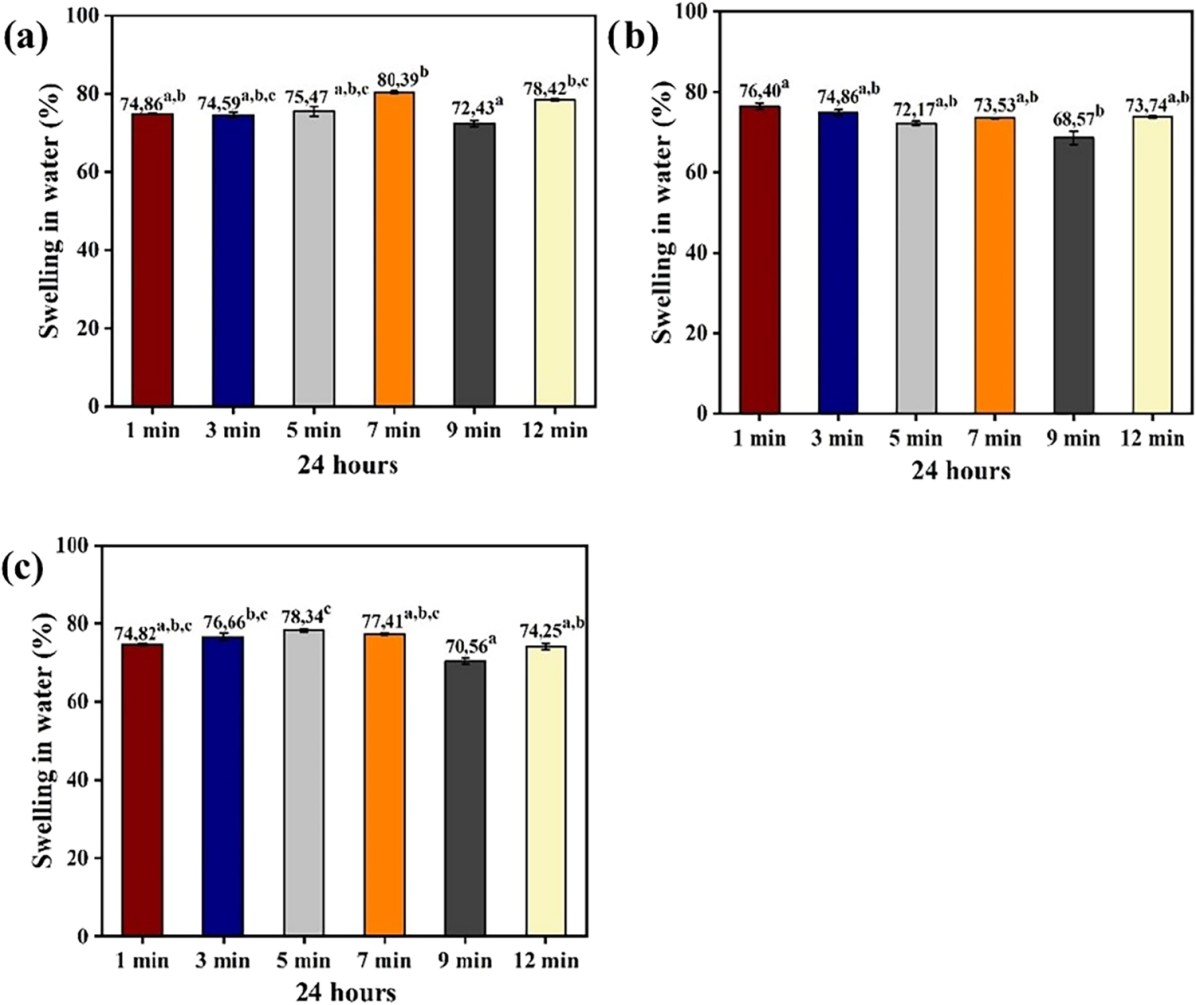
Swelling in water for arrowroot starch films with (a) 25%, (b) 30%, and (c) 35% glycerol at different curing times.

The results for determining the ideal curing time indicated that for all samples containing CA, a curing time of 9 min resulted in the lowest degree of swelling. These results indicate greater stability of the film when subjected to an aqueous medium, demonstrating the success of cross-linking with CA. These findings demonstrate the importance of the ideal curing time for cross-linking. Kumar et al., report that the addition of CA increases the water resistance of the films.^32^ The bonds present in the molecular structure of the acid and starch lead to the establishment of a network of cross-links, contributing to a more resistant structure. Thus, the degree of cross-linking directly influences the swelling capacity.^32^ The compositions with the best performance in terms of swelling and curing time were selected for investigation of tensile, structural, morphological, and degradation properties. Table 2 summarizes the descriptions and compositions of each sample.

**Table 2:** Description, Composition, and Curing Conditions

sample	composition	curing time, and temperature (**°**C)
TPA-25/CA-0	2 g of arrowroot, 0.5 g of glycerol and 60 mL of water	
TPA-25/CA-12	2 g of arrowroot, 0.5 g of glycerol, 0.24 g of CA and 60 mL of water	9 min, 120 °C
TPA-30/CA-0	2 g of arrowroot, 0.6 g of glycerol and 60 mL of water	
TPA-30/CA-9	2 g of arrowroot, 0.6 g of glycerol, 0.18 g of CA and 60 mL of water	9 min, 120 °C
TPA-35/CA-0	2 g of arrowroot, 0.7 g of glycerol and 60 mL of water	
TPA-35/CA-5	2 g of arrowroot, 0.7 g of glycerol, 0.1 g of CA and 60 mL of water	9 min, 120 °C

### 3.3 Cross-Linking Density

The quantitative evolution of the cross-linking density in the TPA films with and without CA reflected significant structural modifications in the macromolecular architecture of the arrowroot starch matrix. The results in Figure 4 show that TPA films cross-linked with CA exhibit a higher density of cross-links than their non-cross-linked analogs in all cycles. These results are inversely proportional to the swelling results in water (Section 3.9). This behavior is expected because the formation of cross-links between starch chains with the aid of CA favors a physical barrier that restricts the diffusion of water molecules into the film.^33^

**Figure 4:**
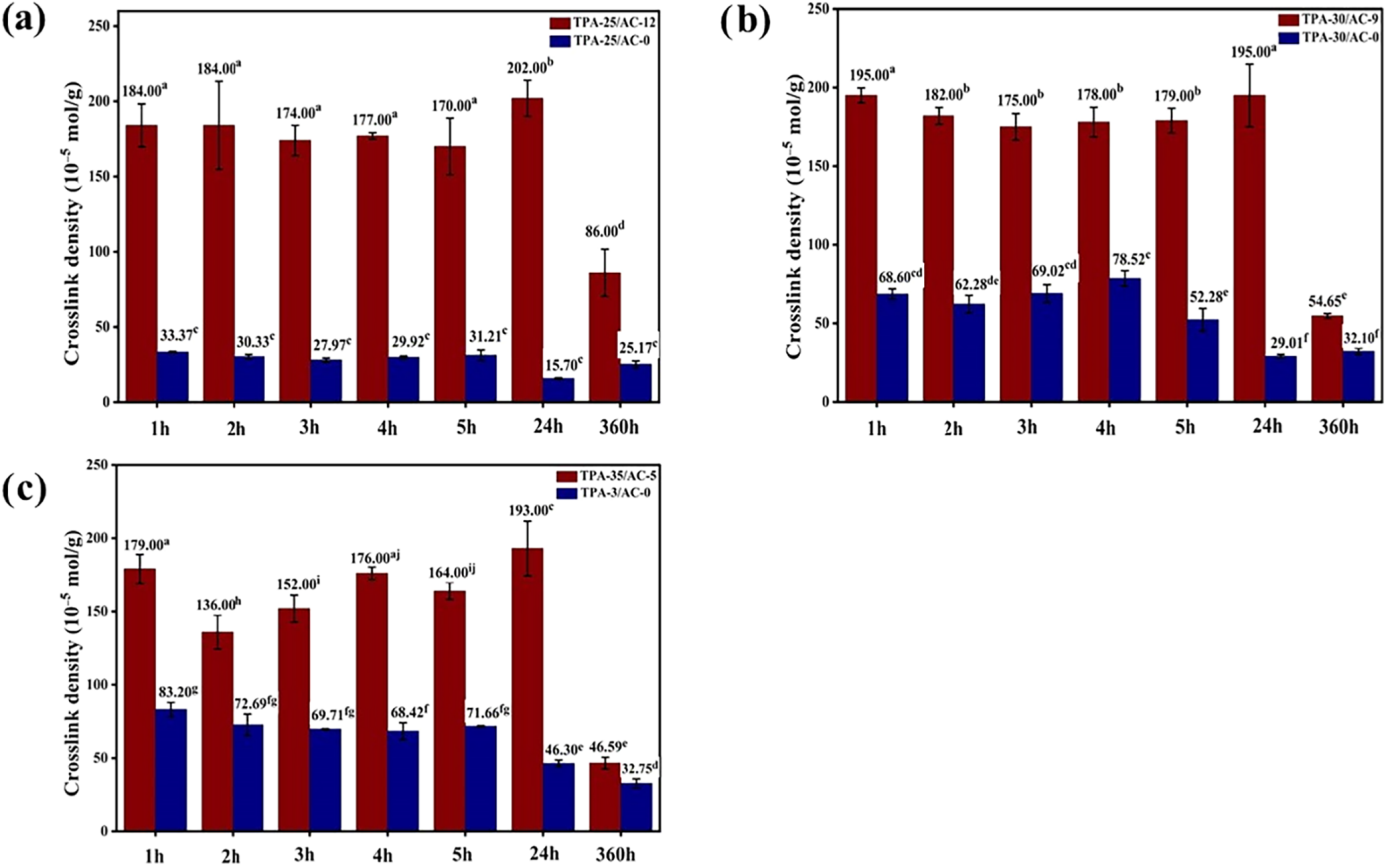
Cross-linking density for (a) TPA-25/CA-0 and TPA-25/CA-12; (b) TPA-30/CA-0 and TPA-30/CA-9; and (c) TPA-35/CA-0 and TPA-35/CA-5 at 1, 2, 3, 4, 5, 24, and 360 h.

### 3.4 Visual Aspect and Thickness

The visual aspect of films intended for packaging production directly influences product acceptance, as their presentation to consumers reveals specific characteristics related to their surface appearance and structure. This relevance is especially pronounced in the food sector, where the film’s transparency allows consumers to clearly visualize the contents, facilitating their purchase decisions.^34^ The visual characteristics of the produced films showed that they had uniform surfaces without bubbles or cracks (Figure 5). All the films produced were odorless and exhibited a degree of transparency. These results are consistent with those obtained for arrowroot starch films that were not cross-linked with CA.^35^ The absence of bubbles or cracks indicates good compatibility between the arrowroot matrix, CA, and glycerol, as well as adequate control of the curing process, ensuring a uniform surface without imperfections. The conformity of these visual characteristics suggests their potential use in biodegradable packaging, as surface uniformity contributes to the structural strength and aesthetics of the final product.

**Figure 5:**
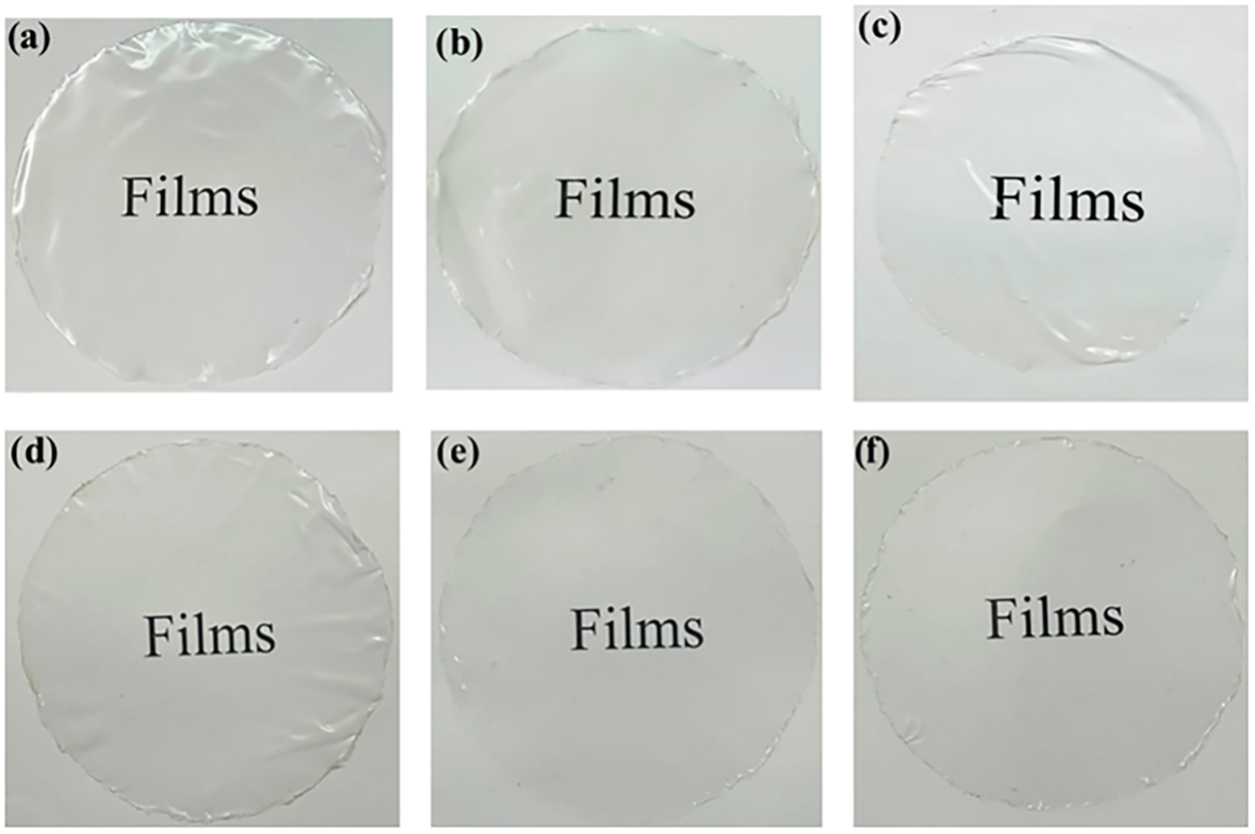
Visual appearance for (a) TPA-25/CA-0; (b) TPA-25/CA-12; (c) TPA-30/CA-0; (d) TPA-30/CA-9; (e) TPA-35/CA-0; (f) TPA-35/CA-5.

The thicknesses of the films did not show significant variations among the different film types (Figure 6). These results indicate that the addition of CA did not significantly change the film thicknesses. Similarly, Pinto et al. reported that the thickness of biodegradable arrowroot starch films remained stable with the addition of ZnO nanoparticles.^36^

**Figure 6:**
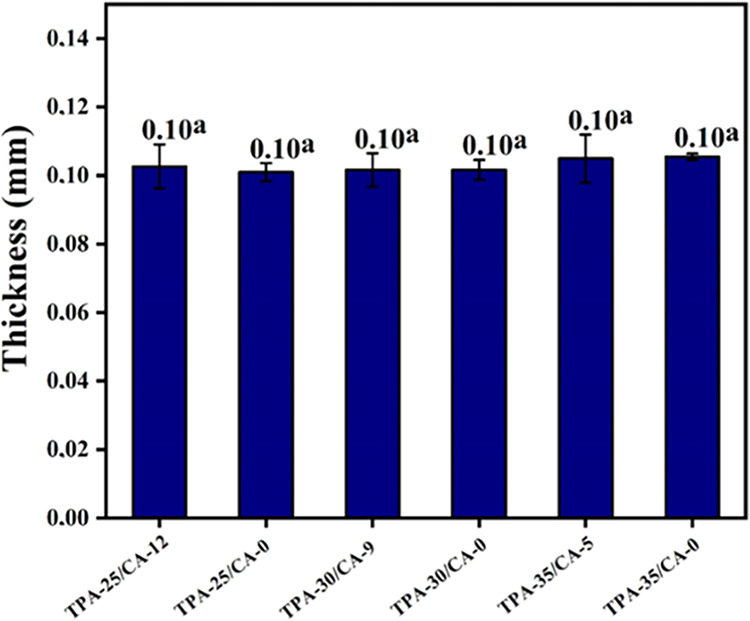
Average thickness for (a) TPA-25/CA-0; (b) TPA-25/CA-12; (c) TPA-30/CA-0; (d) TPA-30/CA-9; (e) TPA-35/CA-0; (f) TPA-35/CA-5.

### 3.5 Fourier Transform Infrared Spectroscopy (FTIR)

FTIR spectroscopy was used to evaluate the chemical interactions between starch, glycerol, and CA in the TPA films. Figure 7 shows the spectra of the films with and without CA. All samples showed a band in the region between 3306 cm^–1^ and 3337 cm^–1^, attributed to the stretching vibrations of the O–H bonds. This band is associated with the hydroxyl groups present in starch and glycerol, as well as the formation of intra- and intermolecular hydrogen bonds.^16,18^ The bands observed at approximately 2900 cm^–1^ are attributed to C–H stretching.^18,37^ The bands observed between 1640 cm^–1^ and 1674 cm^–1^, were attributed the vibrations to the stretching of the H–O–H bonds of the absorbed water and the stretching vibrations of the glycerol hydroxyl groups.^24,38,39^ In the films with CA, bands between 1720 cm^–1^ and 1759 cm^–1^ were observed. These bands are attributed to the stretching vibrations of carbonyl (C=O) groups of esters, indicating the formation of ester bonds between the OH groups of starch and the carboxylic groups of CA.^40,41^ This behavior is characteristic of cross-linking reactions promoted by CA and heat treatment. For all samples, bands between 1022 and 1030 cm^–1^ were observed, which are characteristic of C–O and C–O–C stretching of the anhydroglucose ring of starch.^9,18,42^ Our results are in good agreement with those reported for CA-cross-linked cornstarch. In this study, CA and curing time promoted starch cross-linking through the formation of ester bonds between the carboxylic groups of CA and the hydroxyl groups of starch.^24^

**Figure 7:**
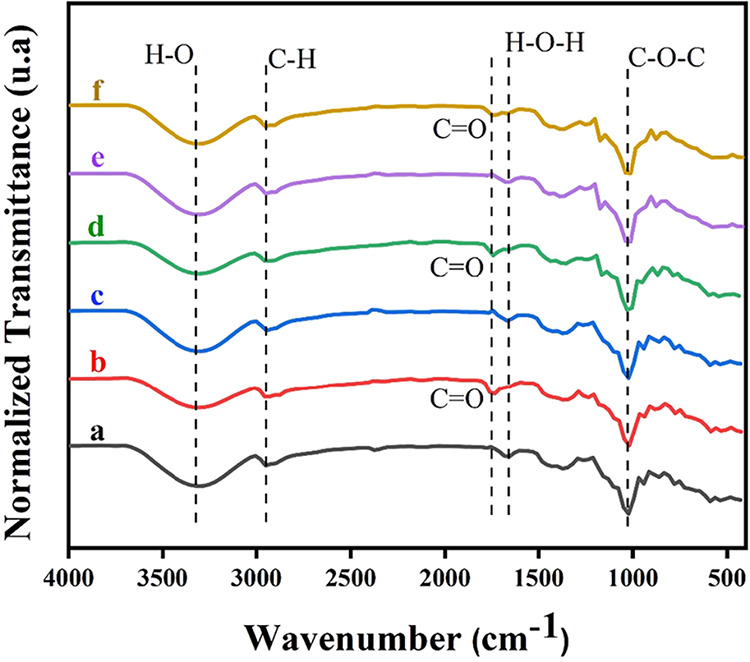
FTIR spectra for (a) TPA-25/CA-0; (b) TPA-25/CA-12; (c) TPA-30/CA-0; (d) TPA-30/CA-9; (e) TPA-35/CA-0; (f) TPA-35/CA-5.

### 3.6 X-ray Diffraction (XRD)

The crystallinity of the TPA films with and without CA were evaluated using XRD. Figure 8 shows the diffractograms for TPA-25/CA-0, TPA-25/CA-12, TPA-30/CA-0, TPA-30/CA-9, TPA-35/CA-0, TPA-35/CA-5. Arrowroot starch has diffraction peaks at 17.26°,19.76°, 20.89°, and 26.85°, characteristic of type A starch.^43^ Additionally, the crystallinity of CA was confirmed by the presence of its characteristic diffraction peaks.^44^ For all samples, broadened peaks were observed at approximately 20°, which is characteristic of the amorphous phase of plasticized starch^23^ These results were also reported by Reddy and Yang, who observed the absence of typical starch peaks at 15° and 23° in films plasticized with glycerol, indicating a lack of crystallinity in the polymers after thermal processing with glycerol.^24^ The prevalence of the amorphous structure confirms that the action of glycerol and shear was effective in deconstructing the granules, as expected during thermoplasticization.^45^ The low-intensity peak for TPA-35/CA-0 at 21.23° suggests the presence of induced crystallinity or retrogradation. This peak may originate from the recrystallization of amylose chains during storage, which occurs more easily in formulations with high plasticizer content.^46,47^ This finding is supported by a study that reported that increasing the glycerol content in corn starch films induced the formation of ordered structures over time. In contrast, TPA-35/CA-5 exhibited a completely amorphous pattern, suggesting that cross-linking may have limited amylose recrystallization. Chi et al., argued that cross-linking with CA provides good resistance to retrogradation because of the strong formation of bonds between starch chains. The addition of CA to TPA-25/CA-12, TPA-30/CA-9, and TPA-35/CA-5 did not alter the characteristic diffraction pattern of arrowroot starch plasticized with glycerol, consistent with previous studies.^2,24^

**Figure 8:**
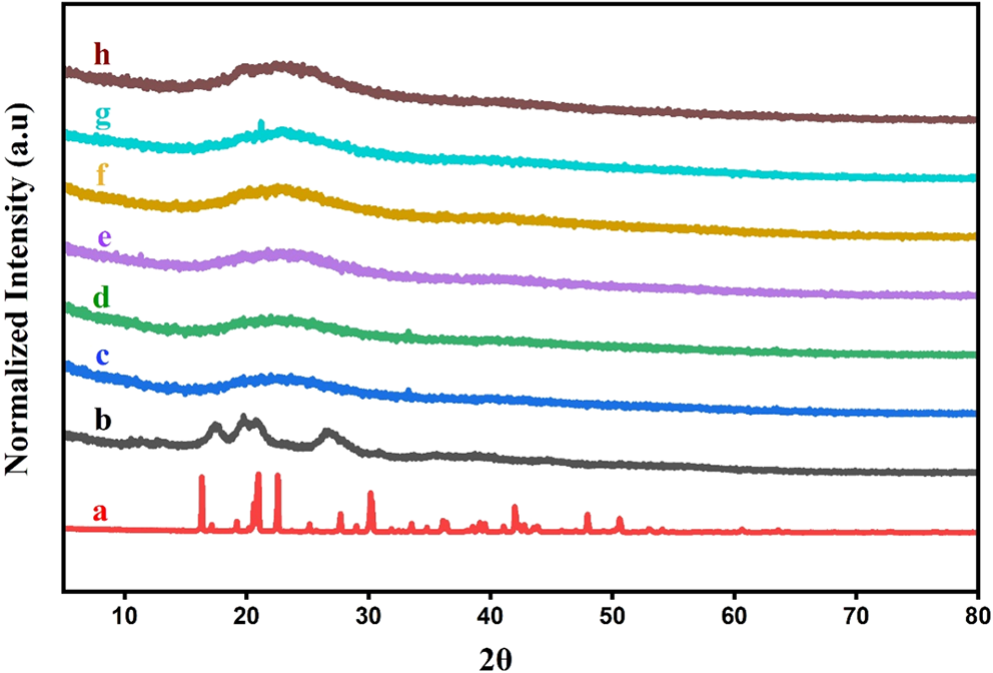
XRD patterns for (a) citric acid; (b) arrowroot starch; (c) TPA-25/CA-0; (d) TPA-25/CA-12; (e) TPA-30/CA-0; (f) TPA-30/CA-9; (g) TPA-35/CA-0; (h) TPA-35/CA-5.

### 3.7 Thermogravimetric Analysis (TGA)

The thermal stability of the TPA films with and without CA was assessed using TGA in an inert nitrogen atmosphere. The TGA and its derivative (DTG) curves are shown in Figure 9. Table 3 shows the temperatures of *T*_10_ (10% mass loss), T50 (50% mass loss), Tmax (maximum temperature of degradation), and residual mass (%) at 700 °C. In general, the TGA results demonstrate that there were three mass loss events for the TPA films with and without CA. The first mass-loss event, below 110 °C, originates from the loss of residual moisture in the films.^48^ The second mass loss for the films occurred in the range of 120–240 °C.^48^ This event was attributed to the moisture loss and thermal decomposition of glycerol. The third event, situated in the 240–390 °C range, is attributed to the thermal decomposition of the polysaccharide chain.^48^ TPA films exhibited a lower *T*_10_ value than CA-cross-linked films. This behavior stems from the cross-linking promoted by CA with the OH groups of the arrowroot starch chains, as confirmed by the FTIR results (Section 3.5), which decrease the moisture content of the films (Section 3.9). Similar results were found for guar gum carboxymethylcellulose films cross-linked with CA, where the authors reported lower *T*_10_ values in the non-CA-cross-linked films.^49^

**Figure 9:**
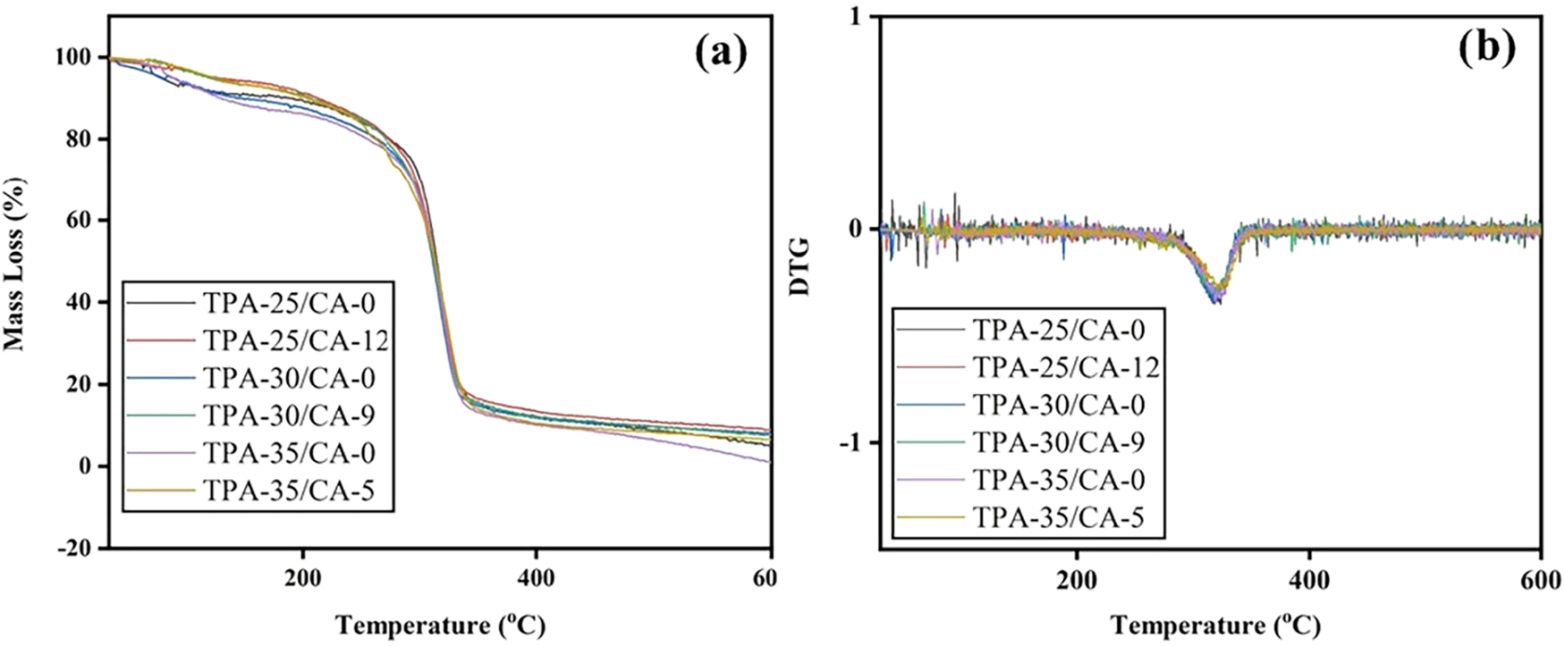
(a) TGA curves for TPA-25/CA-0; TPA-25/CA-12; TPA-30/CA-0; TPA-30/CA-9; TPA-35/CA-0; and TPA-35/CA-5, (b) DTG curves for TPA-25/CA-0; TPA-25/CA-12; TPA-30/CA-0; TPA-30/CA-9; TPA-35/CA-0; and TPA-35/CA-5.

**Table 3:** *T*_10_, *T*_50_, *T*_max_ and Residual Mass for TPA Films with and without CA

sample	*T*_10_ (°C)	*T*_50_ (°C)	*T*_max_ (°C)	residual mass (%) at 600 °C
TPA-25/CA-0	189	315	323	5.11
TPA-25/CA-12	213	314	318	9.02
TPA-30/CA-0	144	312	318	8.07
TPA-30/CA-9	207	313	322	7.56
TPA-35/CA-0	130	313	320	1.00
TPA-35/CA-5	203	314	325	6.47

Our TGA results revealed that TPA films with and without CA exhibited the same thermal decomposition profile in the analyzed temperature range under inert conditions. The films cross-linked with CA had a higher *T*_10_ value than their non-CA cross-linked counterparts. These results support the cross-linking promoted by CA between starch chains, which reduces the final moisture content of the films.

### 3.8 Scanning Electron Microscopy (SEM)

SEM images were acquired to evaluate the cross-sectional morphology of the films, as shown in Figure 10. For the TPA films without CA (Figure 10a–c), surfaces with various discontinuities, such as cracks and porosity, were observed. These defects were more evident in the film with the highest glycerol content, indicating that excess plasticizer may compromise the structural cohesion of the matrix.^46,50^ The absence of chemical cross-linking contributes to the fragility of the material because the interactions present are predominantly physical and reversible, such as hydrogen bonds.^51^ The films cross-linked with CA (Figure 10d–f) exhibited a more regular and continuous morphology. The TPA-25/CA-12 film exhibited a compact microstructure, virtually without cracks, suggesting that the formation of bonds between CA and starch chains favored a more cohesive material structure. Similarly, the TPA-30/CA-9 film also presented a more homogeneous surface with few fissures. In contrast, the TPA-35/CA-5 film showed visible fissures, which may be associated with an imbalance due to the high glycerol content that compromised the polymeric integrity.^35^ This balance between the plasticizer and cross-linking agent is essential for the development of biodegradable materials with a more stable structure, which favors their application as biodegradable packaging.^50,52^

**Figure 10:**
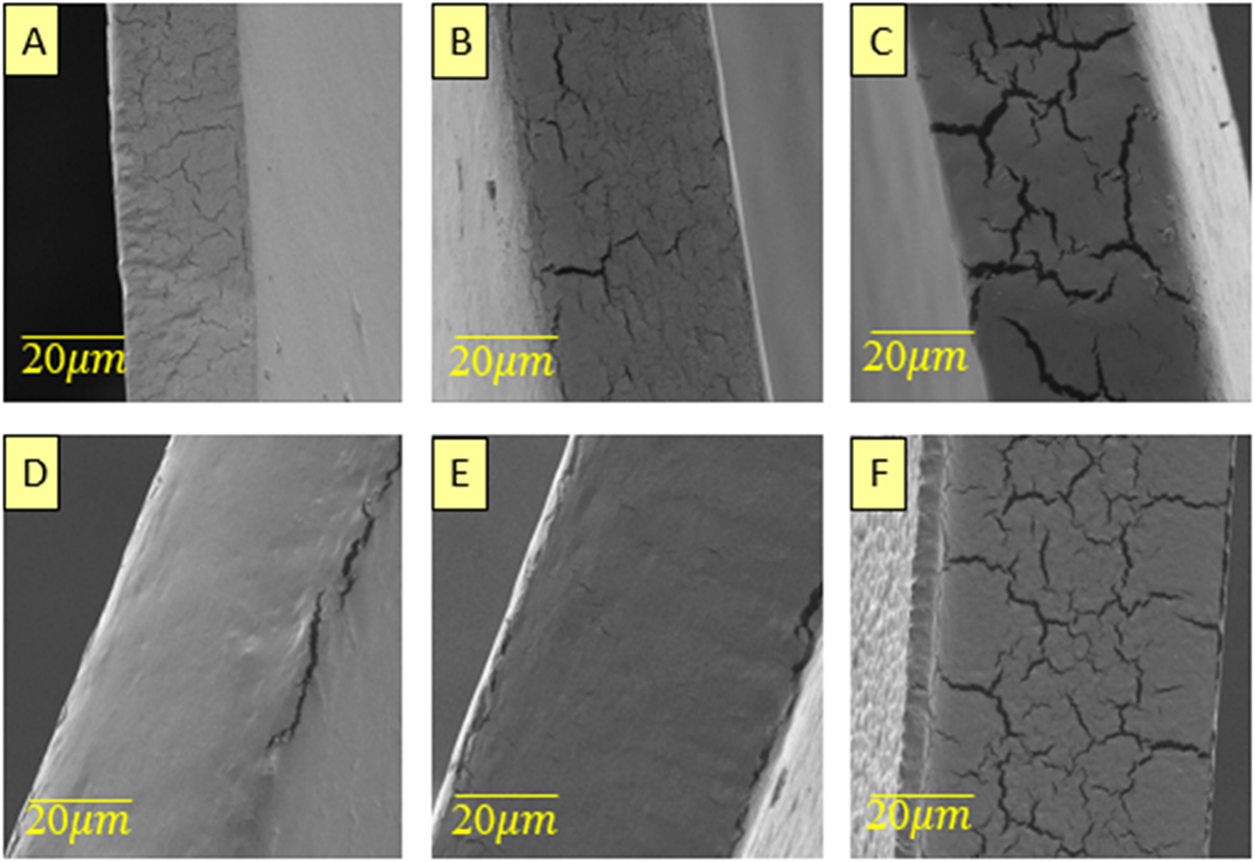
Sem images for (a) TPA-25/CA-0; (b) TPA-30/CA-0; (c) TPA-35/CA-0; (d) TPA-25/CA-12; (e) TPA-30/CA-9; (f) TPA-35/CA-5.

### 3.9 Moisture Content, Water Solubility, Water Vapor Permeability, and Swelling in Water

Moisture content is an important parameter for evaluating the stability and shelf life of biodegradable polymers in food packaging.^2^ Figure 11a shows the results for the moisture content. In our study, the moisture content of the TPA films without CA gradually increased with increasing glycerol concentration. This behavior is associated with the number of hydroxyl groups present in the plasticizer, which contributes to the water absorption in the film matrix.^9,53^ The films containing CA had a lower moisture content than those without CA. This reduction can be attributed to the esterification reaction between the carboxyl and hydroxyl groups of CA and starch, forming a denser polymeric network that hinders water retention.^26,54,55^ Similar results were found for starch films cross-linked with CA.^2^ The MC values for TPA-25/CA-12, TPA-30/CA-9, and TPA-35/CA-5 reveal a reduction in moisture compared to the values for TPA-25/CA-0, TPA-30/CA-0, TPA-35/CA-0, indicating that cross-linking with CA also decreased the hygroscopic nature of the films.

**Figure 11:**
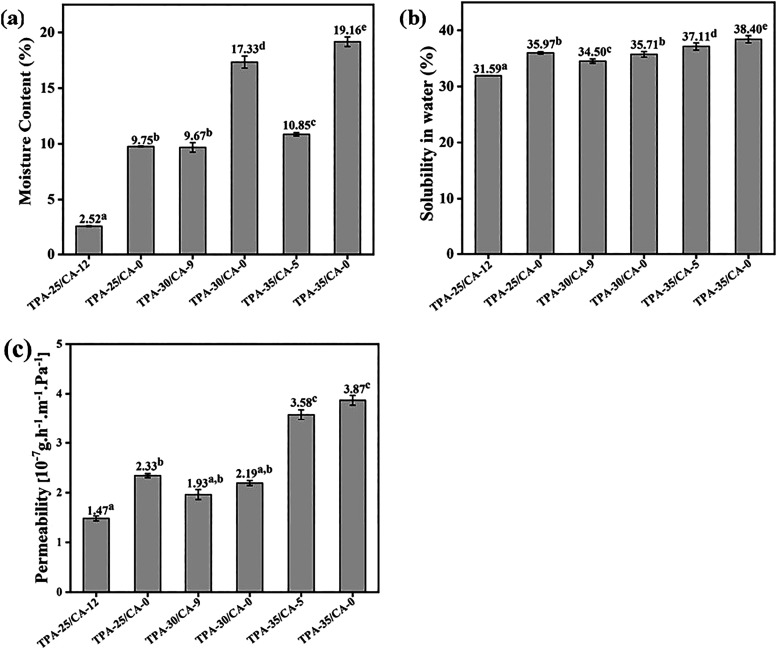
(a) moisture content; (b) solubility in water; and (c) water vapor permeability for TPA-25/CA-0; TPA-25/CA-12; TPA-30/CA-0; TPA-30/CA-9; TPA-35/CA-0; and TPA-35/CA-5.

Water solubility is an important factor in assessing water resistance capacity.^38^ The solubility results are shown in Figure 11b. For films without CA, an increase in solubility was observed, consistent with the increase in glycerol content. These results are attributed to the hydrogen bonds established between the arrowroot starch chains and glycerol molecules, which favor the entry of water molecules, contributing to increased solubility.^56^ Lower solubility values were obtained for the TPA films with CA. This trend is related to the cross-linking that occurs between the acid and starch, which hinders the entry of water into the film matrix, reducing its water absorption capacity.^2,38,54,57^ Similar behavior was reported for rice starch cross-linked with CA.^58^ Thus, our results indicate that the addition of CA tends to decrease the solubility of TPA films.

Water vapor permeability (WVP) is a critical characteristic of films used in biodegradable packaging, as it regulates moisture transfer between food and the external environment, directly influencing the shelf life of the product.^10,54,59,60^ In biodegradable polymers, this property depends on the polymer’s chemical structure, degree of crystallinity, and the presence of additives, which determine the ease with which water molecules pass through the matrix.^10,54^ The water vapor permeation results for the TPA films with and without CA are shown in Figure 11c. For the TPA-25/CA-0 and TPA-25/CA-12 films, a statistically significant difference was observed, which was associated with cross-linking with CA. Cross-linking with CA promotes the formation of a denser polymeric matrix, which, in turn, hinders the passage of water molecules. These results suggest that CA contributed to the formation of a more compact matrix that was less susceptible to the passage of water vapor. For TPA-30/CA-0 and TPA-30/CA-9, the average values for water vapor permeation were statistically similar. The same trend was observed for TPA-35/CA-0 and TPA-35/CA-5. These results may be associated with the low CA content used in the cross-linking and the high glycerol content compared to the permeation results for films with 25% glycerol. The absence of a statistically significant difference in the water vapor permeability (WVP) between the 30% and 35% glycerol cross-linked films and their respective non-cross-linked controls can be explained by the competitive correlation between polymer plasticization and cross-linking. At higher plasticizer concentrations, the distance between the starch chains increases, which increases the internal free volume and allows the passage of water molecules^33^

Our results for the TPA-25/CA-12, TPA-30/CA-9, and TPA-35/CA-5 films are promising compared to those of other films derived from natural polymers (Table 4). Cassava starch films with *Litsea cubeba* essential oil,^61^ had a moisture content of 24.37 ± 4.96 and a water solubility of 29.68 ± 1.53% due to the hydrophilicity of the non-cross-linked starch. Similarly, our results were lower than those of carboxymethylcellulose films with eucalyptus nanocellulose at a moisture content of 21.92%.^62^ However, similar values of moisture and solubility were also observed for the modified corn starch/CMC films,^63^ while starch elastomer films reinforced with silicon dioxide exhibited a higher moisture content (20.53%) and solubility (34.76%).^64^ Carboxymethylcellulose films with eucalyptus^62^ nanocellulose and corn starch/CMC films^63^ exhibited permeation values similar to those obtained in this study. In contrast, starch elastomer films reinforced with silicon dioxide had a higher WVP value of 21.32 × 10^–7^ g·h^–1^·m^–1^·Pa^–1^.^64^

**Table 4:** Moisture Content, Water Solubility, and Water Vapor Permeability of Biodegradable Films

material	moisture content (%)	water solubility (%)	water vapor permeability
Cassava starch/Litsea cubeba essential oil^61^	24.37	29.68	1.11 g·mm·m^–2^·d^–1^·KPa^–1^
Starch/acetylated xylan^65^	21.6	75.48	24.95 g/h·m^2^
Corn starch/Carboxymethyl Cellulose^63^	10.94	36.46	2.52 × 10^–7^ g·h^–1^·m^1^·Pa^–1^
Carboxymethylcellulose/eucalyptus nanocellulose^62^	21.92	27.82	2.78 × 10^–7^g·h^–1^·m^1^·Pa^–1^
Starch elastomers/silicon dioxide^64^	20.53	34.76	21.32 × 10^–7^g·h^–1^·m^1^·Pa^–1^

The results of the swelling in water for TPA films with and without CA are depicted in Figure 12. Figure 12a shows that TPA-25/CA-0 films after immersion in water for all immersion times compared to TPA-25/CA-12 films. TPA-25/CA-12 showed a swelling of 71.99% in the first hour, 72.04% in the second, 72.57% in the third, 72.34% in the fourth, 72.82% in the fifth hour, and after 24 h, the percentage stabilized at 71.01%, reaching 79.08% after 360 h. On the other hand, TPA-25/CA-O exhibited values of 85.75%; 86.32%; 86.78%; 86.40%; 86.16%; 89.75% and 87.38% after 1, 2, 3, 4, 5, 24, and 360 h, respectively. This trend was observed for TPA films with and without CA containing 30% and 35% glycerol (Figure 12b,c). The results revealed that the addition of CA decreased the swelling index of the films with 25, 30, and 35% glycerol, with a more pronounced effect in the first 24 h. This trend is consistent with the findings of Imoisili and Jen, who correlated the reduction in hydrophilicity with cross-linking, contributing to the formation of a more compact polymer network that limits water absorption.^66^ In films with higher glycerol concentrations, swelling tended to decrease more sharply after 360 h. A comparative analysis of glycerol contents at 25%, 30%, and 35% indicates that achieving an ideal balance between plasticization and cross-linking is fundamental for consolidating the network. High concentrations of plasticizer expand the internal free volume, increasing the distance between the polysaccharide chains, making it difficult for citric acid to establish covalent bonds, leading to a gradual decrease in the effective cross-linking density as glycerol content increases.^67,68^ The swelling values for TPS-25/CA-12 and TPS-30/CA-9 reinforce that the addition of CA reduced swelling at the beginning and after 360 h of immersion of the films in water. These data suggest that the inclusion of CA promotes the formation of a more stable structure, making it less susceptible to water damage.

**Figure 12:**
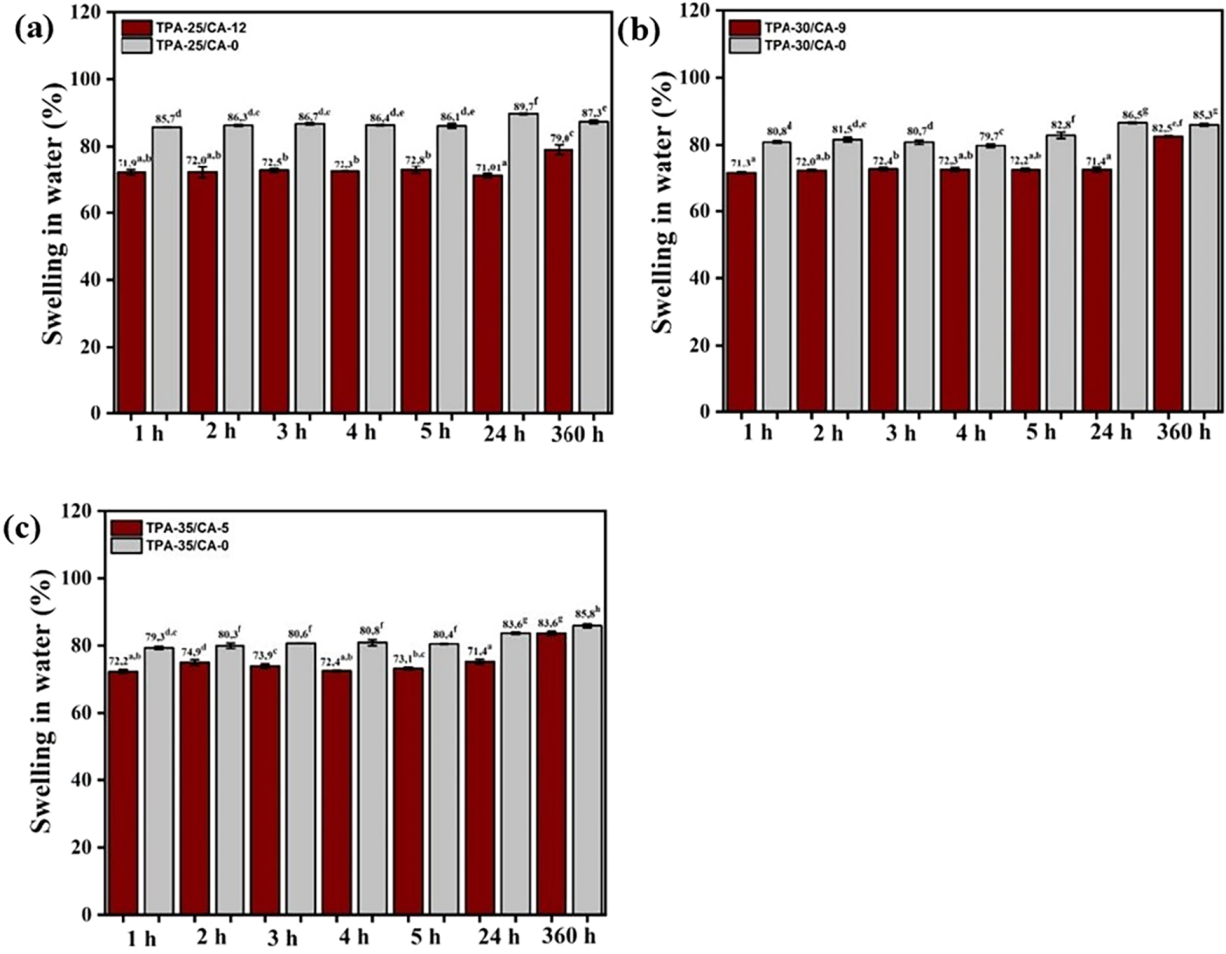
Swelling in water for (a) TPA-25/CA-0 and TPA-25/CA-12; (b)­TPA-30/CA-0 and TPA-30/CA-9; (c) TPA-35/CA-0 and TPA-35/CA-5 at 1, 2, 3, 4, 5, 24, and 360 h. Means followed by equal letters did not differ (*p* < 0.05) by Duncan’s test.

### 3.10 Transparency

Film transparency is important for food packaging applications, as it allows for visual monitoring of the food stored within the films.^69^ Figure 13 shows the average transparency values for the TPA films with and without CA. No statistically significant variation was observed in the transparency of any of the films evaluated. This behavior is interesting because it favors the use of these materials in the food packaging sector by providing adequate monitoring of the visual aspect of foods stored in these films.^70^ In contrast to our results, the addition of CA to potato starch/chitosan films has been reported to promote yellowing.^17^ Thus, our results demonstrate that the cross-linking promoted by CA did not significantly interfere with the optical appearance of the TPA films, suggesting that it is consistent with their visual appearance.

**Figure 13:**
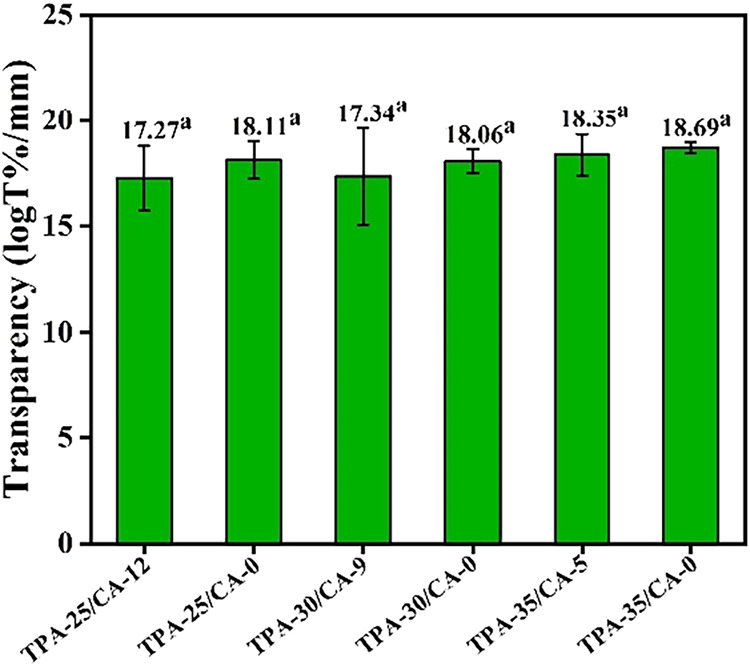
Transparency results for TPA-25/CA-0; TPA-30/CA-0; TPA-35/CA-0; TPA-25/CA-12; TPA-30/CA-9; and TPA-35/CA-5.

### 3.11 Soil Burial Degradation

In this study, soil burial degradation was performed to assess the behavior of films over time when exposed to a natural environment.^2,71^ The films were buried in the soil and monitored on days 2, 3, 5, 7, 10, 12, 14, 16, 18, and 20, and the results displayed in Figure 14. In general, the films showed no signs of degradation after 12 days; however, the films without CA showed greater degradation over time. The TPA films without CA demonstrated a progressive loss of structural integrity, notably after 12 days, with greater intensity for TPA-35/CA-0. This result may be associated with the greater affinity of glycerol for moisture due to its hygroscopic nature, which favors the penetration of water into the polymeric matrix, accelerating hydrolysis and microbial biodegradation.^66,72^ The films containing CA showed subtle signs of degradation in the first few days, with greater evidence after 14 days. These results show that cross-linking with CA promotes the formation of a more cohesive network that is more resistant to moisture and microorganisms.^24,38,71^ However, after 16 d of burial in soil, degradation was more evident in all samples, particularly the complete degradation of TPA-35/CA-0. The slower degradation of the cross-linked films is consistent with the findings of Seligra et al., who attributed this behavior to the cross-linking between starch and CA.^73^ According to the authors, water diffusion in biodegradable polymers causes swelling, which influences microbial growth and the rate of biodegradation. Thus, the presence of CA reduced moisture absorption, resulting in a delay in the onset of degradation. This phenomenon was also reported by Maiti et al., who observed the preservation of film structure for up to 15 days.^74^ Thus, our results demonstrate that the reticulated films exhibited visible integrity for up to 16 d of exposure to soil. Thus, our results show that cross-linking with CA exerts a profound influence on the soil degradation of TPA films.

**Figure 14:**
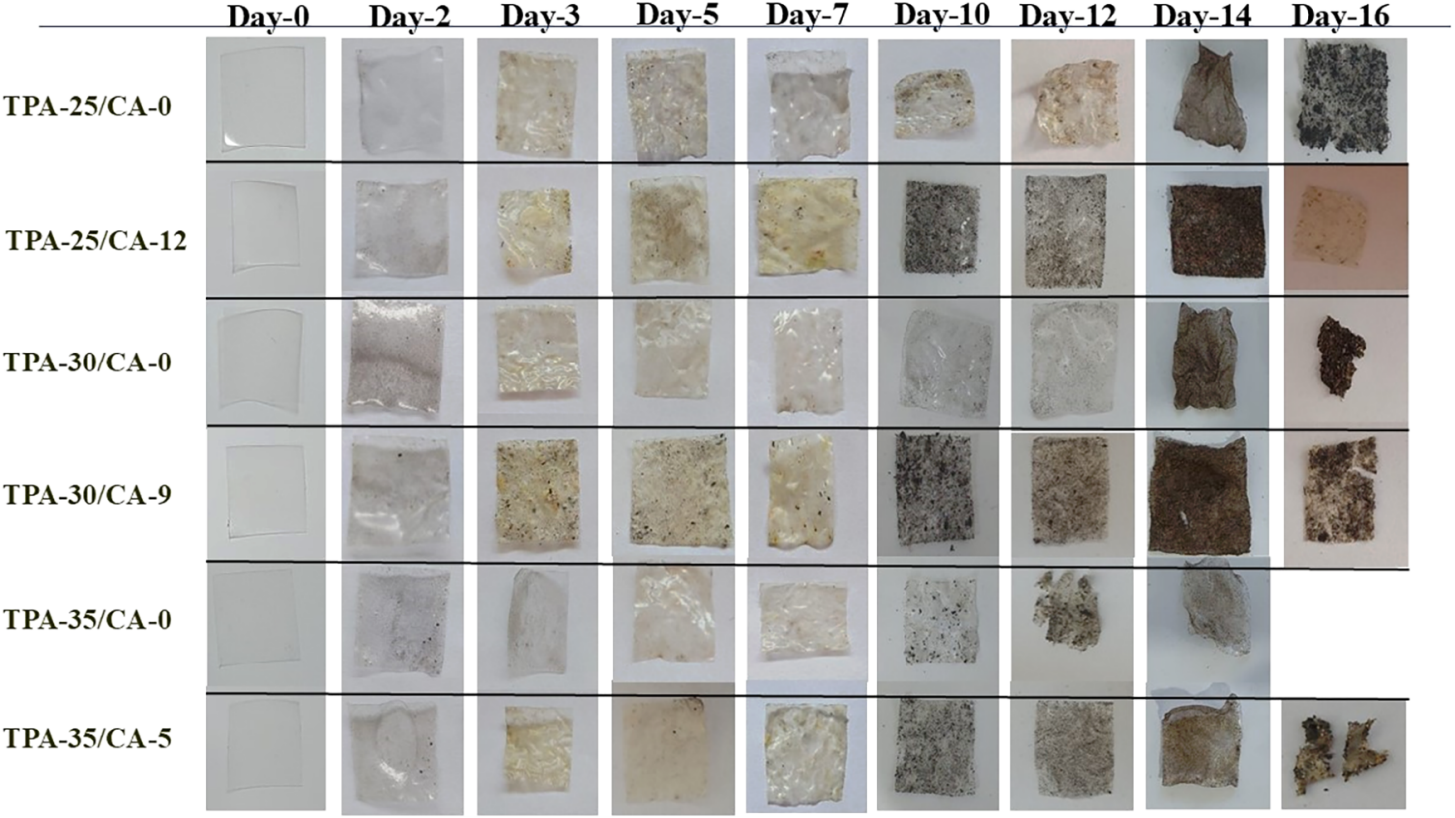
Soil burial degradation for TPA-25/CA-0; TPA-30/CA-0; TPA-35/CA-0; TPA-25/CA-12; TPA-30/CA-9; TPA-35/CA-5 in 0, 2, 3, 5, 7, 10, 12, 14, and 16 days (left to right).

### 3.12 Application as Bread Packaging

The films, before and after cross-linking with CA, were used in a pilot study on bread storage. Figure 15 shows the evaluation of the films stored on day 0 and after 7 days of storage. Bread stored in commercial packaging showed the greatest macroscopic fungal growth among all groups after 7 days. This behavior can be attributed to the passive barrier properties of conventional materials. Commercial packaging usually has a very high water vapor barrier; therefore, the moisture that naturally evaporates from the food is retained, increasing the humidity and generating condensation on the surface of the bread.^75,76^ The bread stored in TPA-25/CA-0, TPA-30/CA-0, and TPA-35/CA-0 films exhibited brown spots on their surface, characterizing the second highest fungal growth rate in the assay. This result indicates that the arrowroot starch matrix, although acting as an initial physical barrier, does not possess properties that can inhibit or delay fungal activity during the evaluation period. Conversely, bread stored in films developed with higher citric acid content, TPA-30/CA-9 and TPA-25/CA-12, exhibited a delay in fungal growth, maintaining the visual integrity of the bread during the evaluation period. Similar findings were reported for bread stored in arrowroot starch and carboxymethylcellulose films cross-linked with citric acid.^75^ Additionally, migration studies are important to assess the impact of the components of these films on consumers because, in addition to technical performance, safety for contact with food is an important requirement. The choice of components classified as Generally Recognized as Safe (GRAS), such as arrowroot starch, glycerol, and citric acid, ensures the safety of the system.^22,23,77^

**Figure 15:**
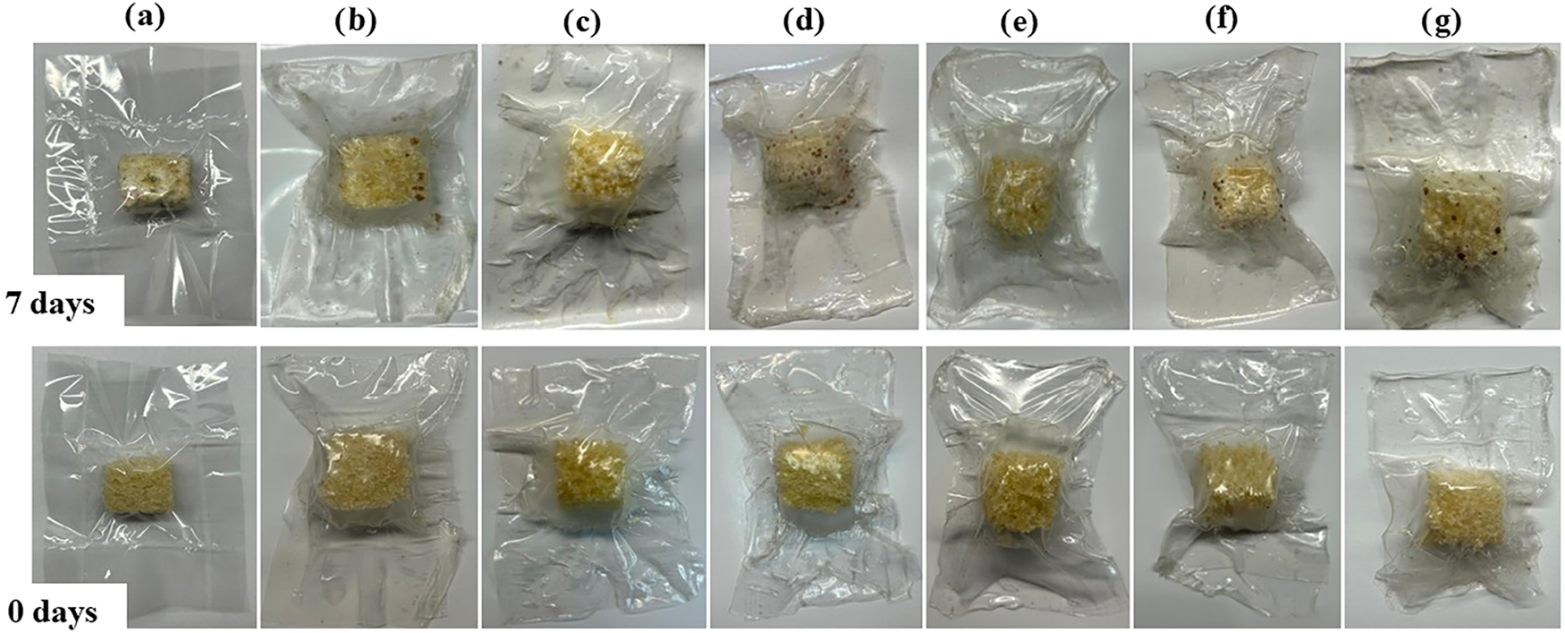
Bread samples packaged in (a) Commercial packaging, (b) TPA-25/CA-0, (c) TPA-25/CA-12, (d) TPA-30/CA-0, (e) TPA-30/CA-9, (f) TPA-35/CA-0, (g) TPA-35/CA-5.

### 3.13 Tensile Properties

The tensile properties of films used in the food packaging sector are fundamental because they demonstrate their behavior when subjected to tensile conditions, such as the handling and transportation of food stored in these films. The results for the tensile strength (σ), elongation (ε), and modulus of elasticity (*E*) are shown in Figure 16. In general, the results for σ demonstrate that the addition of CA promoted an increase in σ. For example, the value of σ for TPA-25/CA-9 was 17.95 MPa, whereas that for TPA-25/CA-0 was 5.28 MPa (Figure 16a). This trend was also observed for TPA films with 30 and 35% glycerol. These findings suggest that CA plays a crucial role in improving the mechanical properties of films, corroborating the results of Skov et al., who highlighted the importance of CA in the formation of cross-links associated with the reinforcement of the polymer matrix.^26^ The reduction in strength observed for films with higher glycerol content indicates that a higher plasticizer content results in greater mobility of the polymer chains, which compromises the intermolecular bonds and consequently, the strength of the material.^8,51^

**Figure 16:**
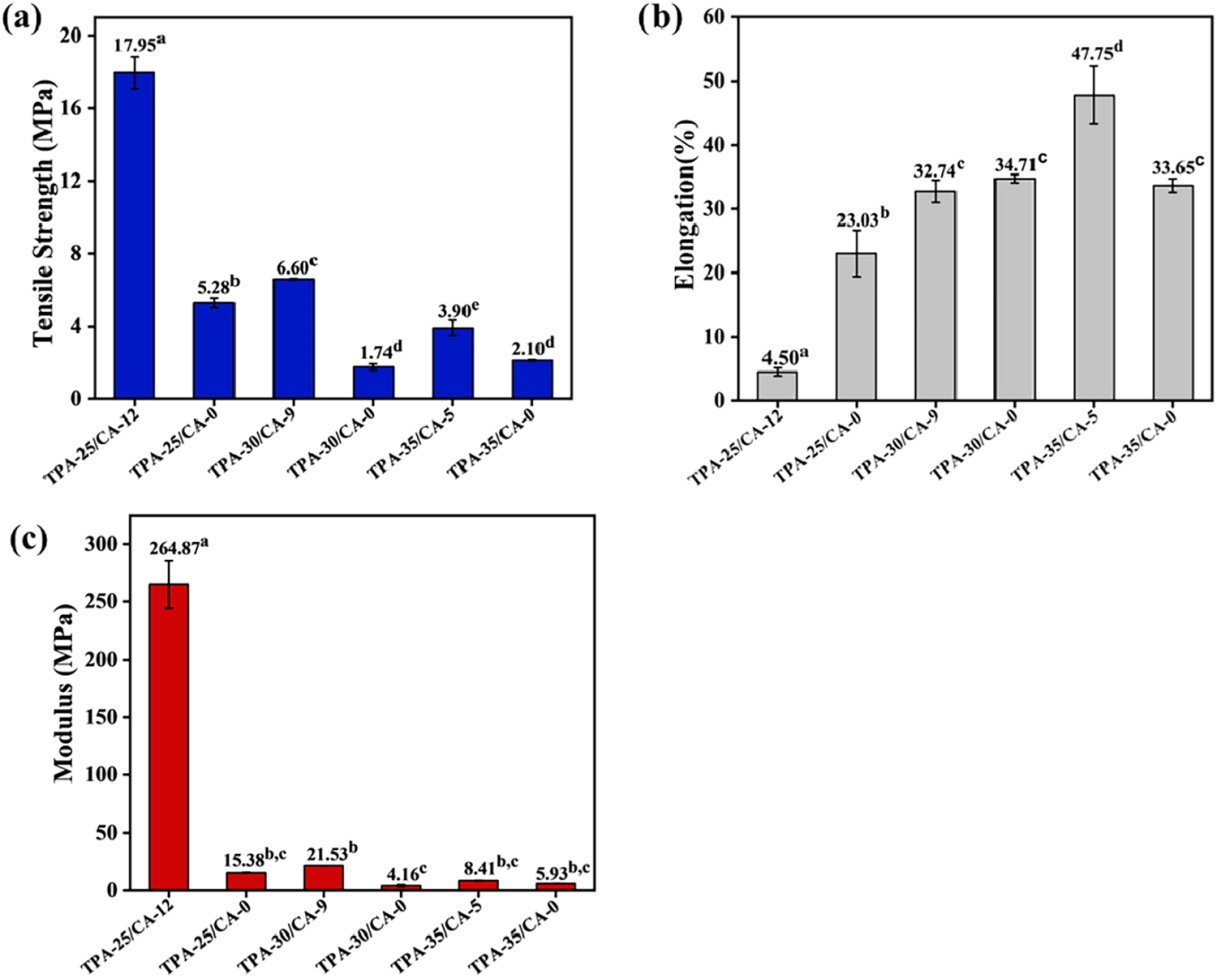
(a) Tensile strength, (b) elongation, and (c) modulus for TPA-25/CA-0; TPA-30/CA-0; TPA-35/CA-0; TPA-25/CA-12; TPA-30/CA-9; and TPA-35/CA-5.

The results from ε revealed values of 4.5% and 23.03% for TPA-25/CA- and TPA-25/CA-12, respectively (Figure 16b). This decrease is attributed to the cross-linking action of CA through the formation of cross-links between the starch chains, producing a more cohesive matrix and thus promoting a decrease in *E*.^26^ This profile was also observed for films with 35% glycerol. Additionally, higher ε values for films with 30 and 35% glycerol are due to the plasticizing action of glycerol, which facilitates mobility between starch chains and thus increases the ε value.^78^ Thus, the results for ε indicate that the addition of CA favors a decrease in the ε values of the TPA films.

For E, the TPA films with CA exhibited a higher modulus than their counterparts without CA, with 264.87 MPa for TPA-25/CA-12 and 15.83 MPa for TPA-25/CA-0, 21.53 MPa for TPA-30/CA-9 and 4.16 MPa for TPA-30/CA-0. As observed in Figure 16c, the high *E* value for TPA-25/CA-12 confirms the action of CA as a cross-linking agent through the formation of cross-links between arrowroot starch chains, forming a more rigid polymeric matrix.^26^ The increase in glycerol content led to a decrease in E, which can be explained by the reduction in the interactions between the chains of the polymer matrix, making it more flexible. No significant differences were found between TPA-35/CA-5 and TPA-35/CA-0, which may be attributed to the high glycerol content used to prepare the films.

The tensile test results for films with different glycerol and CA contents clearly show that mechanical strength, ductility, and modulus of elasticity are strongly influenced by the additives used in the preparation of these films.^8,79,80^ Our results demonstrated that TPA-25/CA-12 exhibited the highest strength and modulus of elasticity. However, this behavior was accompanied by low deformation, which resulted in greater stiffness and brittleness of the material. On the other hand, sample TPA-35/CA-5 showed high deformation but was associated with low strength and lower modulus, which limits its application potential. In this context, TPA-30/CA-9 exhibited a balanced behavior with satisfactory strength, modulus, and elongation. Although PLA films exhibit high strength values such as 2700 MPa, these materials exhibit an elongation close to 9%, resulting in rigid matrices that are prone to fracture under bending stresses.^81^ Chitosan/PVA films containing silver nanoparticles also exhibited this same behavior with σ values between 29 and 46 MPa and ε ranging from 1.6 to 3.9%.^82^ The same trend was observed for corn starch/sisal nanofiber films.^83^ In contrast, the TPA-30/CA-9 film combines adequate mechanical strength to withstand tensile and transport stresses with the flexibility to package food. In addition, the possible interaction between glycerol and citric acid was considered during the interpretation of the results, as glycerol contains hydroxyl groups that could potentially compete with starch hydroxyl groups during the esterification/cross-linking process promoted by citric acid. Although this interaction was not directly quantified in the present study, the obtained results suggest that cross-linking occurred effectively under the selected processing conditions. This can be inferred from the observed changes in the physicochemical and mechanical properties of the films after CA incorporation. Thus, based on the results of swelling in water, soil burial degradation, application as bread packaging and tensile properties, the TPA-30/CA-9 film presents appropriate potential for use in the food packaging sector.

## 4 Conclusion

In this study, TPA films cross-linked with CA were successfully obtained using the solvent casting method, resulting in films with smooth surfaces, free of bubbles and cracks. The results demonstrate that the cross-linking of arrowroot starch with CA, combined with the control of glycerol content and curing time, exerts a decisive influence on the structural, mechanical, and barrier properties of biodegradable films. The formation of ester bonds between arrowroot starch and CA, confirmed by FTIR (1720–1759 cm^–1^), promoted a more cohesive polymer network, as reflected by a significant increase in tensile strength. For films with 30% glycerol, cross-linking increased the tensile strength from 1.74 MPa (TPA-30/CA-0) to 6.60 MPa (TPA-30/CA-9). The XRD results showed broad peaks at approximately 20° for all films, which is characteristic of the amorphous phase of starch. Additionally, cross-linking with CA improved water resistance, as evidenced by reduced solubility, moisture, swelling, and water vapor permeability, without compromising the optical transparency of the films, which is desirable for food packaging. Although cross-linking with CA delayed the initial degradation in soil, all films maintained biodegradable behavior within timeframes consistent with biodegradable materials. The possible interaction between glycerol and citric acid was considered during the interpretation of the results, as glycerol contains hydroxyl groups that could potentially compete with starch hydroxyl groups during the esterification/cross-linking process promoted by citric acid. Although this interaction was not directly quantified in the present study, the obtained results suggest that cross-linking occurred effectively under the selected processing conditions. This can be inferred from the observed changes in the physicochemical and mechanical properties of the films after citric acid incorporation. Bread stored with TPA-30/CA-9 and TPA-25/CA-12, exhibited a delay in fungal growth, maintaining the visual integrity of the bread during the evaluation period. Thus, our results show that arrowroot starch films cross-linked with CA exhibit improved performance and represent a promising and sustainable alternative for the biodegradable packaging of bread. Based on the results of swelling in water, soil burial degradation, bread preservation, and tensile properties, the TPA-30/CA-9 film presents the appropriate potential for use in the food packaging sector.
